# Phlorizin, an Important Glucoside: Research Progress on Its Biological Activity and Mechanism

**DOI:** 10.3390/molecules29030741

**Published:** 2024-02-05

**Authors:** Tongjia Ni, Shuai Zhang, Jia Rao, Jiaqi Zhao, Haiqi Huang, Ying Liu, Yue Ding, Yaqian Liu, Yuchi Ma, Shoujun Zhang, Yang Gao, Liqian Shen, Chuanbo Ding, Yunpeng Sun

**Affiliations:** 1 College of Traditional Chinese Medicine, Jilin Agriculture Science and Technology College, Jilin 132101, China; nitongjia2002@163.com (T.N.); 13778267884@163.com (J.R.); a340211@126.com (J.Z.); ysbz15259920014@126.com (H.H.); 13694423686@163.com (Y.L.); 13674496108@163.com (Y.D.); a18043828340@163.com (Y.L.); 2 College of Chinese Medicinal Materials, Jilin Agricultural University, Changchun 130118, China; zhangshuai4389@163.com; 3 Jilin Aodong Health Technology Co., Ltd., Yanbian 133700, China; zsjmyc999@163.com; 4 Jilin Aodong Yanbian Pharmaceutical Co., Ltd., Yanbian 133700, China; xiaohang0130@163.com; 5 Jilin Jianwei Natural Biotechnology Co., Ltd., Linjiang 134600, China; gaoyang1986516@126.com (Y.G.); shenliqian@126.com (L.S.)

**Keywords:** phlorizin, extraction and identification, biological activity, mechanism

## Abstract

Phlorizin, as a flavonoid from a wide range of sources, is gradually becoming known for its biological activity. Phlorizin can exert antioxidant effects by regulating the IL-1β/IKB-α/NF-KB signaling pathway. At the same time, it exerts its antibacterial activity by reducing intracellular DNA agglutination, reducing intracellular protein and energy synthesis, and destroying intracellular metabolism. In addition, phlorizin also has various pharmacological effects such as antiviral, antidiabetic, antitumor, and hepatoprotective effects. Based on domestic and foreign research reports, this article reviews the plant sources, extraction, and biological activities of phlorizin, providing a reference for improving the clinical application of phlorizin.

## 1. Introduction

Phlorizin is the glucoside of phlorizin, which belongs to the dihydrochalcone class of flavonoids [1]. The leaves of the tea pear are rich in dihydrochalcones, which are traditionally used in the treatment of liver diseases. They can be used as an alternative hepatoprotective agent to prevent and treat liver damage caused by acetaminophen (APAP). Dihydrochalcones from Lithops polystachyon prevent cisplatin-induced nephrotoxicity in mice via mitogen-activated protein kinase pathway-mediated apoptosis. Phlorizin is mainly found in the young leaves, rhizomes, and apple fruits of apples, sweet tea, and other tissues [2]. In addition, phlorizin has multiple health-promoting effects, including cardiovascular protection [3], antiviral [4], antioxidant [5], and antidiabetic activity. Multiple studies have found that phlorizin can inhibit the sodium–glucose transporter system (SGLT1) on the small intestinal epithelial mucosa [6] and block intestinal glucose absorption by improving insulin sensitivity and glucose uptake [7,8]. In addition, phlorizin has a good inhibitory effect on tyrosinase and can prevent certain skin diseases [9]. Therefore, this article reviews the sources, extraction methods, and biological activities of phlorizin to provide a theoretical basis for subsequent research on phlorizin.

## 2. Plant Sources and Extraction Methods of Phlorizin

The main natural source of phlorizin is Malus plants, specifically apple leaves. Apple leaves are a by-product of apple fruit production and show promise as a source for extracting phlorizin [10]. Phlorizin is primarily found in non-edible parts of the apple tree, such as leaves, branches, root bark, seeds, and immature fruits [11,12,13]. Among these parts, the highest concentration of phlorizin is found in the fruit. Apple leaves contain significant amounts of phloretin and its glycoside phlorizin, ranging from 5.4% to 14% of the leaf’s dry weight. The phlorizin content in leaves is not greatly influenced by factors like apple variety or harvest period, making it relatively stable over time and across different apple varieties. Phlorizin is a flavonoid compound that is naturally present in the human diet [14]. It is also found in other food sources like apples, tea, red wine, onions, pomegranate fruit, polygonum, peaches, plums, canine roses, grapes, cranberries, and crabapples (Table 1) [15,16,17].

Phlorizin, as a dihydrochalcone compound, is insoluble in ether and cold water, but can be dissolved in ethanol and hot water. Phlorizin is extracted in a similar manner to other phenolic compounds. Compared with traditional 35% and 80% ethanol extraction, ultrasonic-assisted aqueous two-phase extraction has significant advantages. The loss of ethanol will be reduced, the extraction rate of phlorizin will be increased, and the proportion of impurities will be low. For phlorizin, nuclear magnetic resonance (NMR), high performance liquid chromatography-electrospray ionization mass spectrometry (ESI/MS), and ESI-MS/MS are generally used for analysis.

Phlorizin is known for its poor solubility in water but high solubility in organic solvents [22]. Traditional methods primarily rely on organic solvent extraction [21]. Nowadays, various methods including ultrasonic-assisted extraction [23], microwave-assisted extraction [24,25], and others are commonly employed. Existing literature suggests several techniques for separating phlorizin such as macroporous resin adsorption [26], chitosan flocculation [27], high-performance centrifugal partition chromatography (HPCPC), polyamide chromatography column separation [28], and extraction methods [29]. In this study, phlorizin was extracted from apple branches using water as the solvent in a 10:1 ratio through two extraction cycles. Open macroporous resin chromatography was then performed, followed by ethanol gradient elution. The crude phlorizin was further purified using medium and low-pressure preparative chromatography with ODS as the filler and a methanol–water (3:7) elution. The concentrated products were combined and recrystallized to obtain the pure phlorizin product. The purity of the sample was confirmed to be over 98% using high-performance liquid chromatography (HPLC) [30].

According to reports, a study compared various methods such as ultrasonic and microwave methods. The study determined the optimal extraction process using L16 (43) orthogonal experimental screening. The process involved using red Fuji apples as raw materials, a 40% ethanol solution as the extraction solvent, a solid–liquid ratio of 1:30, and carrying out reflux extraction for 1 h. This resulted in the best extraction effect. Xu [31] achieved a high purity of 98.2% by using high-speed countercurrent chromatography (HSCCC) to isolate and purify phloretin from fruit tree bark. HPCPC technology is widely used in the separation and purification of natural products due to its versatility, low operating cost, high efficiency, rapidity, and high recovery rate. Different methods can be employed to separate and purify phlorizin with higher purity. Researchers can choose the method that best suits their own experimental purpose.

## 3. Study on the Pharmacological Activity of Phlorizin

Phlorizin has a variety of biological activities, which are gradually being understood (Table 2).

### 3.1. Antioxidant Effect

In recent years, extensive research has been conducted on the pharmacological activity of phlorizin, particularly in exploring its potential pharmacological mechanism through its antioxidant effects. The antioxidant effect of phlorizin has been widely studied and is considered an important aspect. The screening of natural bioactive substances with anti-aging properties is currently a trending research topic. Chen et al. [32] discovered that phlorizin increased antioxidant enzyme activity and significantly reduced malondialdehyde content. Through its antioxidant effects, phlorizin can improve the biochemical indicators of aging mice, regulate apoptosis-related proteins to inhibit apoptosis, and modulate the IL-1β/IKB-α/NF-KB signaling pathway to exert antioxidant effects. Wang et al. [15] found that phlorizin, at least partially, alleviated oxidative stress, DNA damage, and apoptosis in HepG2 cells induced by H_2_O_2_ by regulating the expression of Nrf2 protein and apoptosis-related genes. Additionally, Wang et al. [44] suggested that the antioxidant and anti-aging effects of phlorizin may be mediated, to some extent, by its collaboration with the endogenous stress defense system.

**Figure 1:**
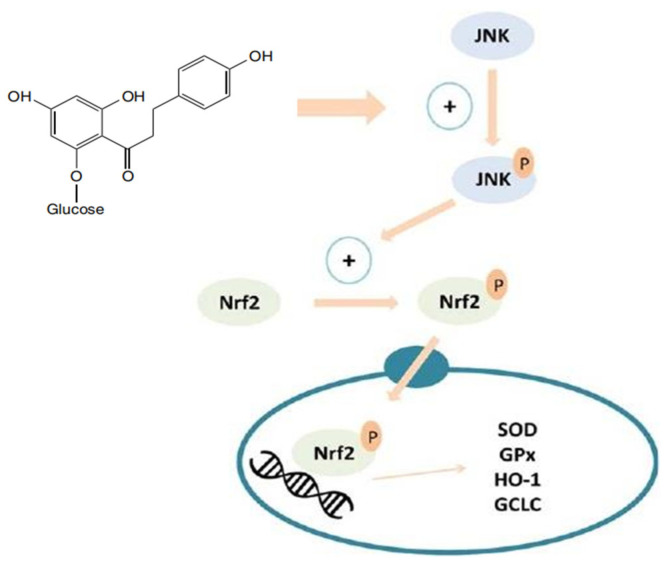
Overview of the mechanism of Nrf2 activation by phlorizin [46].

Oxidative stress is a common feature of many diseases, including cardiovascular disease, cancer, and diabetes. Studies have shown that phlorizin can increase the activity of antioxidant enzymes in the body, such as superoxide dismutase (SOD), catalase (CAT), glutathione peroxidase (GSH-Px), glutathione Glypeptide peroxidase (GPx), and glutathione reductase (GR). This leads to a reduction in oxidative stress in PC12 cells induced by malondialdehyde (MDA) and d-galactose, resulting in a delay in aging [45]. By activating these enzymes, harmful free radicals accumulated in the body can be effectively removed, maintaining intracellular redox balance and reducing cell damage caused by oxidative stress. Furthermore, phlorizin has been found to inhibit various oxidative stress-related signaling pathways, including NF-κB, peroxisome proliferator-activated receptor γ (PPARγ), and NADPH oxidase (NOX) [46]. Through these mechanisms, phlorizin can inhibit inflammatory responses and apoptosis, thereby protecting cells from oxidative stress damage. Molecular docking studies have also revealed that phlorizin can bind to the Keap1 protein, which interacts with the Nrf2 protein. Activation of Nrf2 leads to its translocation to the nucleus and binding to the ARE region of target genes. This activation is partially mediated by JNK phosphorylation. The expression of SOD, GPx, HO-1, and GCLC proteins is regulated by Nrf2 (refer to Figure 1 for more details) [46]. In summary, phlorizin, as a plant compound with important pharmacological activities, exhibits significant antioxidant capacity and cytoprotective effects. It achieves this through the regulation of antioxidant enzyme activity, inhibition of oxidative stress-related signaling pathways, and other mechanisms.

### 3.2. Antibacterial Effect

Previous studies have reported on the bacterial inhibitory activity of phlorizin. Phlorizin has demonstrated the ability to inhibit various Gram-positive and Gram-negative bacteria, including Staphylococcus aureus, Listeria monocytogenes, and Salmonella typhimurium. However, the specific inhibitory mechanism of phlorizin against bacteria remains unclear. In their study, Zhao et al. [33] concluded that phlorizin exhibits the ability to inhibit the growth of Listeria monocytogenes. This suggests that phlorizin may possess strong antibacterial activity against Listeria monocytogenes. The inhibitory mechanism of phlorizin on bacteria is believed to involve intracellular DNA agglutination, reduced intracellular protein and energy synthesis, and disruption of intracellular metabolism.

Electrospun wound dressings serve multiple functions in skin wound care, including antibacterial, anti-inflammatory, and treatment functions. Previous studies have shown that phlorizin can effectively inhibit Staphylococcus aureus RN4220 biofilm formation by up to 70% [47]. Interestingly, the application of CuS nanoparticles to wounds not only increases the incidence of bacterial colonization but also promotes wound healing through re-epithelialization and collagen deposition. It is speculated that phlorizin holds great potential in wound repair, possibly by inhibiting the activation of the target of the rapamycin (mTOR) signaling pathway and enhancing autophagy [22]. In conclusion, phlorizin nanofibers are highly promising materials for wound dressings in future clinical applications, as they meet various requirements of the wound healing process, possess antibacterial effects, and demonstrate potential as effective candidates.

### 3.3. Antiviral Effect

Phlorizin has been found to have antiviral effects. In a study by Zhang et al. [34], the effect and mechanism of phlorizin on bovine viral diarrhea virus (BVDV) infection were experimentally investigated. BVDV is a highly significant pathogen that affects the global cattle industry, and animals infected with BVDV exhibit strain-specific B and T cell immune tolerance [48]. The study explored the underlying mechanism and found that phlorizin inhibits CP BVDV-induced beclin-1 levels and the conversion rate of LC3B-I to LC3B-II. Both in vivo and in vitro experiments demonstrated that phlorizin can effectively inhibit NCP BVDV infection. This discovery highlights the potential of phlorizin as a new dietary strategy for controlling BVDV infection.

Shih-Chao Lin et al. [35] discovered that phlorizin hinders Zika virus infection by interfering with cellular glucose. Zika virus (ZIKV) is a resurfacing flavivirus connected with microcephaly and other neurological diseases [49]. The findings revealed that phlorizin significantly decreased the infection titers of two ZIKV virus strains, namely the African genotype (MR766) and the Puerto Rican genotype (PRVABC59). The decrease in viral yield was attributed to inhibition targeted at the host, which includes reduced activity of apoptotic caspase-3 and -7, as well as diminished phosphorylation of the Akt/mTOR pathway. In conclusion, the study demonstrated the inhibitory impact of phlorizin on ZIKV transmission, and the potential of phlorizin and its ability to inhibit glucose uptake may serve as a valuable foundation for the development of antiviral drugs against ZIKV.

### 3.4. Anti-Diabetic Effect

Phlorizin, the first discovered SGLT inhibitor in the world, selectively and competitively inhibits SGLT1 and SGLT2, leading to increased urinary glucose excretion and reduced blood glucose levels [50,51]. Research has shown that phlorizin can significantly improve blood sugar levels and lipid metabolism in diabetic mice [52]. It regulates intestinal flora and renal glucose absorption, lowers blood sugar, restores normal blood sugar levels, increases insulin sensitivity, and improves dyslipidemia in diabetic animal models. Streptozotocin (STZ) is commonly used as a diabetogenic drug in animal models of diabetic nephropathy (DN) [37]. Its mechanism is similar to that of phlorizin, as it has an insulin-like effect and directly acts on HMG-CoA reductase to reduce cholesterol synthesis. It is also associated with inhibiting phosphodiesterase, reducing cAMP decomposition, and inhibiting triacylglycerol secretion [53]. Studies have revealed significant differences in the pharmacokinetic behavior of phlorizin between normal rats and STZ-induced type 2 diabetes (T2D) rats. The AUC value and Cmax of T2D rats are higher compared to normal rats, while the T1/2 value is lower. Furthermore, the bioavailability of phlorizin and its metabolites is higher in T2D rats than in normal rats [36,54].

Phlorizin has demonstrated a significant impact on mitigating the complications associated with diabetes. These complications often include cardiovascular and cerebrovascular diseases, kidney damage, and neuropathy. Through various mechanisms, phlorizin can effectively reduce the harm caused by these complications. Zhang et al. [37] conducted a study using a high-fat diet to induce diabetes in rats and treated them with intraperitoneal injections of STZ. The results showed that phlorizin treatment improved the symptoms of diabetes. Additionally, phlorizin exhibited the ability to lower fasting blood sugar, improve blood lipid levels, protect pancreatic islets from damage, and reduce fat accumulation in liver cells. Furthermore, phlorizin was found to safeguard the vision of C57 BLKS/J db/db mice by interfering with retinal neuropathy, vascular proliferation, and apoptosis following oxidative stress. This protective mechanism may be associated with the inhibition of glycogen synthase kinase-3 by phlorizin, as well as pathway activation, regulation of the ubiquitin-proteasome pathway, and reduction in the production of advanced glycation end-products (AGEs) [39]. Although phlorizin has been employed in diabetes treatment for over a century, its precise molecular mechanisms have not been fully elucidated. Lu et al. [38] discovered that phlorizin significantly reduces weight gain and levels of blood glucose, total cholesterol (TC), and triglycerides (TG), shedding light on the essential insights into the mechanism of phlorizin in treating diabetes, particularly in the liver.

The aforementioned research demonstrates that phlorizin has the potential to effectively lower blood sugar levels and decrease dependency on insulin, which holds significant importance for managing diabetes. Moreover, phlorizin exhibits the ability to prevent and ameliorate diabetes-related complications, as well as enhance diabetes management through the regulation of energy metabolism and weight control. Conducting further research will facilitate a comprehensive understanding of the mechanisms underlying phlorizin’s action, advance its clinical application, and offer more efficacious treatments for individuals with diabetes.

### 3.5. Anti-Tumor Effect

Phlorizin, a widely used treatment for various diseases, has been found to have anti-tumor effects. Esophageal cancer is a common malignant tumor of the digestive tract, ranking 7th in morbidity and 6th in mortality among global malignant tumors according to 2018 global cancer statistics [40]. While neoadjuvant therapy [55,56], minimally invasive surgery, and modern precision radiotherapy [40] have improved survival rates for esophageal cancer in recent years, the overall efficacy remains low. Numerous studies have demonstrated the significant role of the JAK/STAT signaling pathway in the growth, metastasis, and apoptosis of esophageal cancer [57,58]. Jia et al. [59] found that phlorizin inhibits cell proliferation, invasion, migration, and autophagy, and activates apoptosis by antagonizing the JAK2/STAT3 signaling pathway. This discovery provides a theoretical basis and potential for utilizing phlorizin as a natural food or pharmaceutical ingredient in the treatment of esophageal cancer.

Numerous studies have demonstrated the inhibitory effects of phlorizin on the growth of various types of cancer cells, including human leukemia, bladder cancer, rat mammary adenocarcinoma, B16 mouse melanoma, and HL60 human leukemia cells [60,61]. However, despite the extensive research on phlorizin’s anti-cancer properties, it is characterized by poor solubility in lipids and water, an unclear target of action, and limited efficacy. In an attempt to address these limitations, Wang et al. [60] synthesized a series of phlorizin derivatives, which exhibited moderate cytotoxicity against several cancer cell lines, such as A549 (human lung cancer), SPC-A1 (human non-small cell lung cancer), EC109 (human esophageal cancer), MCF-7 (human breast adenocarcinoma), and MDA-MB-231 (human breast cancer). These findings highlight the potential of phlorizin and its derivatives as effective inhibitors of cancer cells. Additionally, Parthasarathi Perumal et al. [62] discovered that phlorizin significantly reduced malignant tumors in mice with liver cancer induced by diethylnitrosamine and CCl4, while also normalizing blood biochemical indicators, thus demonstrating its promising in vivo anti-cancer activity.

**Figure 2:**
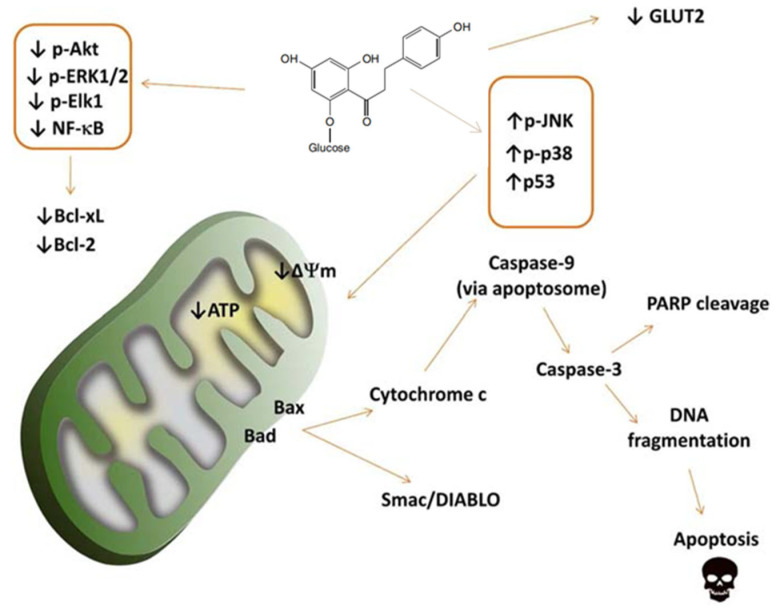
The pro-apoptotic pathway activated by phlorizin in tumor cells [63].

According to research, the activation of pro-apoptotic protein kinases (JNK and p38) and upregulation of p53 lead to the release of cytochrome c and Smac/DIABLO proteins from mitochondria to the cytosol. In the cytosol, cytochrome c activates caspase-9 by binding to apoptotic bodies, which in turn activates caspase-3. Caspase-3 cleaves PARP, responsible for DNA repair. DNA fragmentation is a hallmark of apoptosis, demonstrating the ability of phlorizin to trigger cell death through intrinsic pro-apoptotic pathways. The downregulation of pro-survival pathways, including Akt and ERK1/2, plays an important role in the anti-tumor effects of phlorizin (Figure 2) [63]. In summary, phlorizin exerts its anti-tumor effect by regulating cell proliferation and apoptosis processes, inhibiting the proliferation of tumor cells. It also regulates the expression of cell cycle-related proteins, blocking the progression of the cell cycle and thereby inhibiting the division and growth of tumor cells. Additionally, phlorizin promotes the apoptosis of tumor cells and inhibits their survival and spread by regulating apoptosis-related signaling pathways. Phlorizin’s ability to regulate cell growth and death is crucial to its anti-tumor effect.

### 3.6. Protecting the Liver

Phlorizin, a nutrient found in apple peel, has been utilized for over 100 years due to its various biological activities, including antioxidant properties and liver protection. Non-alcoholic fatty liver disease (NAFLD) is a prevalent chronic liver disease in Western countries [64]. NAFLD encompasses a wide range of pathophysiological conditions, with inflammation being a characteristic feature of nonalcoholic steatohepatitis (NASH), which can potentially progress to cirrhosis and hepatocellular carcinoma [65,66]. NAFLD is considered as the hepatic component of obesity-related metabolic syndrome and type 2 diabetes (T2D), which is characterized by impaired glucose metabolism [67]. Studies have shown that improvement in glucose metabolism is accompanied by the amelioration of NAFLD, leading to the reversal of steatosis and cystosis, and a reduction in the expression of hepatic lipogenic enzymes [68]. The short-term use of the dual SGLT1/2 inhibitor, phlorizin, can restore glycemic control and hepatic glucose metabolism, significantly improving NASH and promoting changes in hepatic glucose metabolism associated with T2D-related NAFLD [43]. It has been reported that phlorizin can alleviate oxidative stress, maintain the activity of endogenous antioxidant enzymes, preserve normal nerve cell morphology, and enhance cognitive ability and memory in D-galactose-induced mice. Furthermore, studies on PC12 cells have suggested that the anti-aging and liver-protective effects of phlorizin may be attributed to its interaction with the Nrf2 pathway [46,69].

The main chemical component of selenium tea (E-Se tea) is phlorizin [45]. Zhang et al. [70] investigated the protective effect and potential mechanism of E-Se tea on acetaminophen (APAP)-induced acute liver injury. Histopathological analysis revealed that phlorizin could suppress APAP-induced inflammatory infiltration, necrosis, and hepatocyte vacuolization. The study also demonstrated that phlorizin increased the expression of antioxidant genes (SOD2, Gpx1, GCLC, and GCLM). These findings indicate that phlorizin can effectively ameliorate liver damage, inhibit inflammatory response, and reduce oxidative stress.

### 3.7. Other Functions

Phlorizin has been reported to have significant inhibitory effects on damage caused by ultraviolet B (UVB) irradiation in nude mice, including erythema, epidermal thickening, and skin shedding. It also reduces the apoptosis of HaCaT cells induced by UVB irradiation. The mechanism behind these effects is believed to be related to the activation of the MAPK/NF-κB apoptosis signaling pathway [71]. Furthermore, phlorizin has been found to enhance the self-repair ability of normal human keratinocytes and fibroblasts. This improvement is thought to be associated with the regulation of the mTOR signaling pathway and the increased expression of vascular endothelial cell growth factors [72]. In vitro studies have demonstrated that phlorizin significantly improves the survival rate of human neuroblastoma cells (SH-SY5Y) or cerebral cortical neurons when exposed to hydrogen peroxide (H_2_O_2_) or high sugar levels (glucose > 17.5 mmol·L^−1^). This effect is attributed to the inhibition of SGLT-mediated Na^+^ and glucose influx [73]. In vivo studies have shown that administering phlorizin to the lateral ventricle can significantly reduce the volume of cerebral infarction in mice induced by middle cerebral artery occlusion (MCAO). The mechanism behind this reduction is associated with the inhibition of SGLT by phlorizin and its anti-apoptotic signaling [74].

Phlorizin has been found to have significant inhibitory effects on ischemic contracture induced by Langendorff cardiac perfusion-induced myocardial ischemia in guinea pigs. It also plays a role in preventing and treating arrhythmias caused by acute global ischemia. The mechanism of action is believed to be related to the regulation of voltage-dependent calcium ion (Ca^2+^) channels and the inhibition of Ca^2+^ influx [75]. Additionally, phlorizin has shown the ability to inhibit symptoms such as weight loss, diarrhea, and hematochezia in mice with acute colitis caused by dextran sulfate sodium. It also improves colon cell morphology and intestinal brush border integrity [76]. Furthermore, intraperitoneal injection of phlorizin has been found to significantly inhibit cell death in the hippocampal CA3 area of male ddY mice induced by bilateral carotid artery occlusion (BCAO), thereby improving learning and memory impairment in mice. This improvement may be attributed to the inhibition of SGLT family genes [77]. Overall, these research findings highlight the beneficial effects of phlorizin in various areas such as skin loss resistance, cerebral ischemia resistance, colitis improvement, myocardial ischemia resistance, and memory enhancement [78,79]. However, further studies are needed to fully understand the underlying mechanisms in action.

## 4. Conclusions and Outlook

The medicinal phlorizin dihydrochalcone demonstrates numerous biological activities, such as antioxidant, antibacterial, antiviral, antidiabetic, antitumor, and hepatoprotective properties. This review offers a comprehensive overview of its common biological activities and potential mechanisms, presenting new opportunities for the utilization of phlorizin. It is important to highlight that the research and development of anti-tumor drugs has garnered significant attention from both domestic and international researchers.

Phlorizin, a dihydrochalcone compound, has a slight sweet taste and can be used as a food additive in the food industry. At the same time, it has good moisturizing properties and strong antioxidant capacity, and it also shows great market application prospects in skin care products. With the deepening of research, the biological activity of phlorizin is gradually being understood. The various biological activities it exhibits have strong appeal in the medical field. Soon, phlorizin may occupy a larger market in the food, cosmetics, and pharmaceutical fields.

In recent years, significant progress has been made in understanding the biological activity mechanism of phlorizin. However, there are still unresolved issues and unexplored areas regarding its application for various diseases. Therefore, future research should focus on deepening our understanding of its mechanism of action, toxic and side effects, and potential clinical applications. This will help promote the development and application of phlorizin in the medical field, making significant contributions to human health.

Phlorizin docosahexaenoate, a novel fatty acid ester of plant polyphenols, inhibits the formation of spheroids in breast cancer stem cells and exhibits cytotoxic effects on paclitaxel-resistant triple-negative breast cancer cells [80,81,82]. It can be seen that the acylated derivatives of phlorizin have good biological activity in anti-cancer. We will pay more attention to research in this area in the future, the development and application of anti-cancer drugs.

The intersection of disciplines promotes the development of various fields now. The antioxidant, antibacterial, and antiviral biological activities of phlorizin are combined with electrospinning technology and medical materials such as hydrogels to expand the application site of phlorizin from the body to in vivo application. These have played a very good guiding role in the utilization of phlorizin and are also important directions for our development and utilization of phlorizin.

## Figures and Tables

**Table 1:** Plant sources of phlorizin.

Serial Number	Plant Sources	Parts	Place of Origin	Reference
1	*Malus pumila* Mill.	Skin, fruit, juice, pulp	Shanxi, Shaanxi, Ningxia, Gansu, and other areas in China	[10]
2	*Punica granatum* L.	pulps	Anhui, Shaanxi, Yunnan, Sichuan, etc., China	[16]
3	*Reynoutria japonica* Houtt.	flower	Shaanxi, Gansu, EastChina, Sichuan, and other areas in China	[18]
4	*Prunus persica* (L.) Batsch	pulps	Gansu, Sichuan, Liaoning, Zhejiang, and other areas in China	[18]
5	*Rosa canina Gremli* ex Chris	pulps	Neighboring regions of Europe, Northern Africa, and Iraq	[19]
6	*Vitis vinifera* L.	pulps	Shandong, Shaanxi, Turpan, and other areas in China	[19]
7	*Vaccinium macrocarpon* Ait.	Pulps	Shandong, Jiangsu, Liaoning, Hebei, and other areas in China	[20]
8	*Malus spectabilis* (Ait.) Borkh.	leaf	Shandong, Henan, Anhui, Jiangsu, and other areas in China	[21]

**Table 2:** Pharmacological activities of phlorizin and its mechanism of action.

Pharmacological Activity	Dosage of Rhizopyranoside	Experimental Model	Mechanism	References
antioxidant	20 and 40 mg/kg	D-galactose (D-gal) aging mouse model	Regulation of apoptosis-related proteins inhibits apoptosis and exerts antioxidant effects by modulating the IL-1β/IKB-α/NF-kB signaling pathway.	[32]
	100 and 150 μg/mL	A model of hydrogen peroxide-induced oxidative damage in HepG2 cells	Regulation of Nrf2 protein and apoptosis-related gene expression to alleviate h2o2-induced oxidative stress, DNA damage, and apoptosis in HepG2 cells.	[15]
antimicrobial	100–200 μg/mL	Listeria monocytogenes model	Causes aggregation of intracellular DNA, leading to reduced protein synthesis.	[33]
antiviral	6.25, 12.5, 25 mg/kg	Mouse models of BVDV infection	Promotes IFN-α and IFN-β levels, decreases IL-1β and IL-6 expression, and regulates rig -1, MDA5, TLR3, and NLRP3 levels.	[34]
	6.25, 12.5, 25, 50 and 100 μM	Zika virus cell model	Reduces apoptotic caspase-3 and -7 activity and reduces phosphorylation of the Akt/mTOR pathway.	[35]
antidiabetic	20 mg/kg	Type 2 diabetes mellitus (db/db) mouse model	Increases abundance of beneficial bacterial communities, inhibits the growth of pathogenic bacteria, reduces LPS loading into the host and increases levels of short-chain fatty acids (SCFAs) in the gut.	[36]
	30, 60 and 120 mg/kg	Streptozotocin (STZ) treatment in a rat model of diabetes mellitus	Decreases FBG levels, decreases serum TC, TG, and LDL-C levels, and increases HDL-C levels.	[37]
	200 mg/kg	Streptozotocin (STZ)-induced diabetes model in sober rats	Inhibition of glycogen synthase kinase-3 pathway activation modulates the ubiquitin proteasome pathway and reduces the production of advanced glycosylation end products (AGEs).	[38,39]
	100 µg/mL	Human KYSE450 and KYSE30 cells were modeled with rmi-1640 medium in 10% bovine fetal bovine serum.	Inhibits the JAK2/stat3 signaling pathway and inhibits the JAK2/stat3 signaling pathway.	[40]
antitumor	10 mg/kg	HepG2 tumor explant mice	Associated with GLUT2, inhibits PKC expression and regulates apoptosis.	[41]
	1–5 μmol/200 μL	A model of skin tumors in mice by fobol ester	Inhibition of TPA blocks the upstream ERK signaling pathway by inactivating NF-jB-induced COX-2 expression.	[42]
protection of the liver	0.4 g/kg	T2D animal model of obesity obtained by injection of monosodium glutamate (MSG)	Resumption of glycemic control and hepatic glucose metabolism and substantial improvement in NASH, reinforcing the involvement of altered hepatic glucose metabolism in T2D-associated NAFLD.	[43]

## References

[1] Dong H., Ning Z., Yu L., Li L., Lin L., Huang J. (2007). Preparative separation and identification of the flavonoid phlorhizin from the crude extract of lithocarpus polystachyus rehd. Molecules.

[2] Zhang J., Tang L., Hu X., Zeng Z., Wu W., Geng F., Li H., Wu D. (2024). Evaluation of the binding affinity and antioxidant activity of phlorizin to pepsin and trypsin. Food Sci. Human Wellness.

[3] Ridgway T., O’Reilly J., West G., Tucker G., Wiseman H. (1997). Antioxidant action of novel derivatives of the apple-derived flavonoid phloridzin compared to oestrogen: Relevance to potential cardioprotective action. Biochem. Soc. Trans..

[4] Gosch C., Halbwirth H., Stich K. (2010). Phloridzin: Biosynthesis, distribution and physiological relevance in plants. Phytochemistry.

[5] Rezk B.M., Haenen G.R., van der Vijgh W.J., Bast A. (2002). The antioxidant activity of phloretin: The disclosure of a new antioxidant pharmacophore in flavonoids. Biochem. Biophys. Res. Commun..

[6] Zhang L., Yang X., Zhang Y., Zhai Y., Xu W., Zhao B., Liu D., Yu H. (2012). Biotransformation of phlorizin by human intestinal flora and inhibition of biotransformation products on tyrosinase activity. Food Chem..

[7] Wang J., Huang Y., Li K., Chen Y., Vanegas D., Mclamore E.S., Shen Y. (2016). Leaf extract from lithocarpus polystachyus rehd. Promote glycogen synthesis in t2dm mice. PLoS ONE.

[8] Kumar S., Sinha K., Sharma R., Purohit R., Padwad Y. (2019). Phloretin and phloridzin improve insulin sensitivity and enhance glucose uptake by subverting ppargamma/cdk5 interaction in differentiated adipocytes. Exp. Cell Res..

[9] Lu L., Zhai X., Li X., Wang S., Zhang L., Wang L., Jin X., Liang L., Deng Z., Li Z. (2022). Met1-specific motifs conserved in otub subfamily of green plants enable rice otub1 to hydrolyse met1 ubiquitin chains. Nat. Commun..

[10] Gao T.H., Liao W., Lin L.T., Zhu Z.P., Lu M.G., Fu C.M., Xie T. (2022). Curcumae rhizoma and its major constituents against hepatobiliary disease: Pharmacotherapeutic properties and potential clinical applications. Phytomedicine.

[11] Bai R., Zhu J., Bai Z., Mao Q., Zhang Y., Hui Z., Luo X., Ye X.Y., Xie T. (2022). Second generation beta-elemene nitric oxide derivatives with reasonable linkers: Potential hybrids against malignant brain glioma. J. Enzym. Inhib. Med. Chem..

[12] Huang B., Gui M., An H., Shen J., Ye F., Ni Z., Zhan H., Che L., Lai Z., Zeng J. (2023). Babao dan alleviates gut immune and microbiota disorders while impacting the tlr4/myd88/nf-small ka, cyrillicb pathway to attenuate 5-fluorouracil-induced intestinal injury. Biomed. Pharmacother..

[13] Zhao Y., Dong Y., Chen X., Wang Z., Cui Z., Ni S. (2023). Using sulfide as nitrite oxidizing bacteria inhibitor for the successful coupling of partial nitrification-anammox and sulfur autotrophic denitrification in one reactor. Chem. Eng. J..

[14] Xiang J., Mlambo R., Shaw I., Seid Y., Shah H., He Y., Kpegah J., Tan S., Zhou W., He B. (2023). Cryopreservation of bioflavonoid-rich plant sources and bioflavonoid-microcapsules: Emerging technologies for preserving bioactivity and enhancing nutraceutical applications. Front. Nutr..

[15] Kan Y., Kan H., Bai Y., Zhang S., Gao Z. (2023). Effective and environmentally safe self-antimildew strategy to simultaneously improve the mildew and water resistances of soybean flour-based adhesives. J. Clean Prod..

[16] Su Y., Wang M., Yang J., Wu X., Xia M., Bao M., Ding Y., Feng Q., Fu L. (2023). Effects of yulin tong bu formula on modulating gut microbiota and fecal metabolite interactions in mice with polycystic ovary syndrome. Front. Endocrinol..

[17] Wang H., Yang T., Wu J., Chen D., Wang W. (2023). Unveiling the mystery of sumo-activating enzyme subunit 1: A groundbreaking biomarker in the early detection and advancement of hepatocellular carcinoma. Transplant. Proc..

[18] Zhang X., Su M., Du J., Zhou H., Li X., Li X., Ye Z. (2019). Comparison of phytochemical differences of the pulp of different peach [*Prunus persica* (l.) Batsch] cultivars with alpha-glucosidase inhibitory activity variations in china using uplc-q-tof/ms. Molecules.

[19] Hvattum E. (2002). Determination of phenolic compounds in rose hip (*Rosa canina*) using liquid chromatography coupled to electrospray ionisation tandem mass spectrometry and diode-array detection. Rapid Commun. Mass Spectrom..

[20] Turner A., Chen S.N., Joike M.K., Pendland S.L., Pauli G.F., Farnsworth N.R. (2005). Inhibition of uropathogenic escherichia coli by cranberry juice: A new antiadherence assay. J. Agric. Food Chem..

[21] Zhang Z., Liu F., He C., Yu Y., Wang M. (2017). Optimization of ultrasonic-assisted aqueous two-phase extraction of phloridzin from malus micromalus makino with ethanol/ammonia sulfate system. J. Food Sci..

[22] Sun S., Hao M., Ding C., Zhang J., Ding Q., Zhang Y., Zhao Y., Liu W. (2022). Sf/pvp nanofiber wound dressings loaded with phlorizin: Preparation, characterization, in vivo and in vitro evaluation. Colloid Surf. B-Biointerfaces.

[23] Liu Y., Liu H., Xia Y., Guo H., He X., Li H., Wu D., Geng F., Lin F., Li H. (2021). Screening and process optimization of ultrasound-assisted extraction of main antioxidants from sweet tea (*Lithocarpus litseifolius* [hance] chun). Food Biosci..

[24] Shang A., Luo M., Gan R., Xu X., Xia Y., Guo H., Liu Y., Li H. (2020). Effects of microwave-assisted extraction conditions on antioxidant capacity of sweet tea (*Lithocarpus polystachyus* rehd.). Antioxidants.

[25] Moreira M.M., Barrosoa M.F., Boeykens A., Withouck H., Morais S., Delerue-Matos C. (2017). Valorization of apple tree wood residues by polyphenols extraction: Comparison between conventional and microwave-assisted extraction. Ind. Crop. Prod..

[26] Sun L., Guo Y., Fu C., Li J., Li Z. (2013). Simultaneous separation and purification of total polyphenols, chlorogenic acid and phlorizin from thinned young apples. Food Chem..

[27] Wang Q., Liu J., Liu X., Zha H., Ren N. (2014). Purification of phloridzin from lithocarpus polystachyus rehd leaves using polymeric adsorbents functionalized with glucosamine and β-cyclodextrin. Chem. Pap..

[28] Tian L., Cao J., Zhao T., Liu Y., Khan A., Cheng G. (2021). The bioavailability, extraction, biosynthesis and distribution of natural dihydrochalcone: Phloridzin. Int. J. Mol. Sci..

[29] Kammerer J., Boschet J., Kammerer D.R., Carle R. (2011). Enrichment and fractionation of major apple flavonoids, phenolic acids and dihydrochalcones using anion exchange resins. Lwt-Food Sci. Technol..

[30] Lijia X., Guo J., Chen Q., Baoping J., Zhang W. (2014). Quantitation of phlorizin and phloretin using an ultra high performance liquid chromatography-electrospray ionization tandem mass spectrometric method. J. Chromatogr. B.

[31] Xue K., Lue H., Que B., Shan H., Song J. (2010). High-speed counter-current chromatography preparative separation and purification of phloretin from apple tree bark. Sep. Purif. Technol..

[32] Chen H., Dong L., Chen X., Ding C., Hao M., Peng X., Zhang Y., Zhu H., Liu W. (2022). Anti-aging effect of phlorizin on d-galactose -induced aging in mice through antioxidant and anti-inflammatory activity, prevention of apoptosis, and regulation of the gut microbiota. Exp. Gerontol..

[33] Zhao P., Zhang Y., Deng H., Meng Y. (2022). Antibacterial mechanism of apple phloretin on physiological and morphological properties of listeria monocytogenes. Food Sci. Technol..

[34] Zhang Z., Huang J., Zhao Z., Yuan X., Li C., Liu S., Cui Y., Liu Y., Zhou Y., Zhu Z. (2022). In vivo and in vitro antiviral activity of phlorizin against bovine viral diarrhea virus. J. Agric. Food Chem..

[35] Lin S.C., Chen M.C., Liu S., Callahan V.M., Bracci N.R., Lehman C.W., Dahal B., de la Fuente C.L., Lin C.C., Wang T.T. (2019). Phloretin inhibits zika virus infection by interfering with cellular glucose utilisation. Int. J. Antimicrob. Agents.

[36] Wang Z., Gao Z., Wang A., Jia L., Zhang X., Fang M., Yi K., Li Q., Hu H. (2019). Comparative oral and intravenous pharmacokinetics of phlorizin in rats having type 2 diabetes and in normal rats based on phase ii metabolism. Food Funct..

[37] Mei X., Zhang X., Wang Z., Gao Z., Liu G., Hu H., Zou L., Li X. (2016). Insulin sensitivity-enhancing activity of phlorizin is associated with lipopolysaccharide decrease and gut microbiota changes in obese and type 2 diabetes (db/db) mice. J. Agric. Food Chem..

[38] O’Neill J., Fasching A., Pihl L., Patinha D., Franzen S., Palm F. (2015). Acute sglt inhibition normalizes o2 tension in the renal cortex but causes hypoxia in the renal medulla in anaesthetized control and diabetic rats. Am. J. Physiol.-Renal Physiol..

[39] Zhang W., Chen S., Fu H., Shu G., Tang H., Zhao X., Chen Y., Huang X., Zhao L., Yin L. (2021). Hypoglycemic and hypolipidemic activities of phlorizin from lithocarpus polystachyus rehd in diabetes rats. Food Sci. Nutr..

[40] Lu W.D., Li B.Y., Yu F., Cai Q., Zhang Z., Yin M., Gao H.Q. (2012). Quantitative proteomics study on the protective mechanism of phlorizin on hepatic damage in diabetic db/db mice. Mol. Med. Rep..

[41] de Oliveira M.R. (2016). Phloretin-induced cytoprotective effects on mammalian cells: A mechanistic view and future directions. Biofactors.

[42] Yang K., Tsai C., Wang Y., Wei P., Lee C., Chen J., Wu C., Ho Y. (2009). Apple polyphenol phloretin potentiates the anticancer actions of paclitaxel through induction of apoptosis in human hep g2 cells. Mol. Carcinog..

[43] Yamazaki Y., Usui I., Kanatani Y., Matsuya Y., Tsuneyama K., Fujisaka S., Bukhari A., Suzuki H., Senda S., Imanishi S. (2009). Treatment with srt1720, a sirt1 activator, ameliorates fatty liver with reduced expression of lipogenic enzymes in msg mice. Am. J. Physiol.-Endocrinol. Metab..

[44] Wang H., Sun Z., Liu D., Li X., Rehman R., Wang H., Wu Z. (2019). Apple phlorizin attenuates oxidative stress in drosophila melanogaster. J. Food Biochem..

[45] Liu Y., Liu Y., Guo Y., Xu L., Wang H. (2021). Phlorizin exerts potent effects against aging induced by d-galactose in mice and pc12 cells. Food Funct..

[46] Bhakkiyalakshmi E., Sireesh D., Rajaguru P., Paulmurugan R., Ramkumar K.M. (2015). The emerging role of redox-sensitive nrf2-keap1 pathway in diabetes. Pharmacol. Res..

[47] Silva N.H.C.S., Garrido-Pascual P., Moreirinha C., Almeida A., Palomares T., Alonso-Varona A., Vilela C., Freire C.S.R. (2020). Multifunctional nanofibrous patches composed of nanocellulose and lysozyme nanofibers for cutaneous wound healing. Int. J. Biol. Macromol..

[48] Peterhans E., Schweizer M. (2013). Bvdv: A pestivirus inducing tolerance of the innate immune response. Biologicals.

[49] Slavov S.N., Otaguiri K.K., Kashima S., Covas D.T. (2016). Overview of zika virus (zikv) infection in regards to the brazilian epidemic. Brazilian J. Med. Biol. Res..

[50] Alvarado F., Crane R.K. (1964). Studies on the mechanism of intestinal absorption of sugars. Vii. Phenylglycoside transport and its possible relationship to phlorizin inhibition of the active transport of sugars by the small intestine. Biochim. Biophys. Acta.

[51] Panayotova-Heiermann M., Loo D.D., Wright E.M. (1995). Kinetics of steady-state currents and charge movements associated with the rat na+/glucose cotransporter. J. Biol. Chem..

[52] Najafian M., Jahromi M.Z., Nowroznejhad M.J., Khajeaian P., Kargar M.M., Sadeghi M., Arasteh A. (2012). Phloridzin reduces blood glucose levels and improves lipids metabolism in streptozotocin-induced diabetic rats. Mol. Biol. Rep..

[53] Brouwers B., Pruniau V.P.E.G., Cauwelier E.J.G., Schuit F., Lerut E., Ectors N., Declercq J., Creemers J.W.M. (2013). Phlorizin pretreatment reduces acute renal toxicity in a mouse model for diabetic nephropathy. J. Biol. Chem..

[54] Katsuda Y., Sasase T., Tadaki H., Mera Y., Motohashi Y., Kemmochi Y., Toyoda K., Kakimoto K., Kume S., Ohta T. (2015). Contribution of hyperglycemia on diabetic complications in obese type 2 diabetic sdt fatty rats: Effects of sglt inhibitor phlorizin. Exp. Anim..

[55] Greally M., Ilson D.H. (2018). Neoadjuvant therapy for esophageal cancer: Who, when, and what?. Cancer.

[56] Borggreve A.S., Kingma B.F., Ruurda J.P., van Hillegersberg R. (2021). Safety and feasibility of minimally invasive surgical interventions for esophageal and gastric cancer in the acute setting: A nationwide cohort study. Surg. Endosc..

[57] Semenkovich T.R., Subramanian M., Yan Y., Hofstetter W.L., Cassivi S.D., Inra M.L., Stiles B.M., Altorki N.K., Chang A.C., Brescia A.A. (2019). Adjuvant therapy for node-positive esophageal cancer after induction and surgery: A multisite study. Ann. Thorac. Surg..

[58] Timme S., Ihde S., Fichter C.D., Waehle V., Bogatyreva L., Atanasov K., Kohler I., Schoepflin A., Geddert H., Faller G. (2014). Stat3 expression, activity and functional consequences of stat3 inhibition in esophageal squamous cell carcinomas and barrett’s adenocarcinomas. Oncogene.

[59] Liu J., Wu W., Liu S., Zuo L., Wang Y., Yang J., Nan Y. (2015). Nimesulide inhibits the growth of human esophageal carcinoma cells by inactivating the jak2/stat3 pathway. Pathol. Res. Pract..

[60] Kobori M., Shinmoto H., Tsushida T., Shinohara K. (1997). Phloretin-induced apoptosis in b16 melanoma 4a5 cells by inhibition of glucose transmembrane transport. Cancer Lett..

[61] Jia Z., Xie Y., Wu H., Wang Z., Li A., Li Z., Yang Z., Zhang Z., Xing Z., Zhang X. (2021). Phlorizin from sweet tea inhibits the progress of esophageal cancer by antagonizing the jak2/stat3 signaling pathway. Oncol. Rep..

[62] Wang L., Li Z.W., Zhang W., Xu R., Gao F., Liu Y.F., Li Y.J. (2014). Synthesis, crystal structure, and biological evaluation of a series of phloretin derivatives. Molecules.

[63] Perumal P., Arthanari U., Sanniyasi E. (2023). Phlorizin isolated from seagrass syringodium isoetifolium inhibits diethylnitrosamine and carbon tetrachloride-induced hepatocellular carcinoma in balb/c mice. S. Afr. J. Bot..

[64] Shin J.W., Kundu J.K., Surh Y.J. (2012). Phloretin inhibits phorbol ester-induced tumor promotion and expression of cyclooxygenase-2 in mouse skin: Extracellular signal-regulated kinase and nuclear factor-kappab as potential targets. J. Med. Food.

[65] Tevar A.D., Clarke C., Wang J., Rudich S.M., Woodle E.S., Lentsch A.B., Edwards M.L. (2010). Clinical review of nonalcoholic steatohepatitis in liver surgery and transplantation. J. Am. Coll. Surg..

[66] Safaei A., Arefi O.A., Mohebbi S.R., Rezaei-Tavirani M., Mahboubi M., Peyvandi M., Okhovatian F., Zamanian-Azodi M. (2016). Metabolomic analysis of human cirrhosis, hepatocellular carcinoma, non-alcoholic fatty liver disease and non-alcoholic steatohepatitis diseases. Gastroenterol. Hepatol. Bed Bench.

[67] Brunt E.M. (2005). Pathology of nonalcoholic steatohepatitis. Hepatol. Res..

[68] Firneisz G. (2014). Non-alcoholic fatty liver disease and type 2 diabetes mellitus: The liver disease of our age?. World J. Gastroenterol..

[69] David-Silva A., Esteves J.V., Morais M.R.P.T., Freitas H.S., Zorn T.M., Correa-Giannella M.L., Machado U.F. (2020). Dual sglt1/sglt2 inhibitor phlorizin ameliorates non-alcoholic fatty liver disease and hepatic glucose production in type 2 diabetic mice. Diabetes Metab. Syndr. Obes..

[70] Medhat E., Rashed L., Abdelgwad M., Aboulhoda B.E., Khalifa M.M., El-Din S.S. (2020). Exercise enhances the effectiveness of vitamin d therapy in rats with alzheimer’s disease: Emphasis on oxidative stress and inflammation. Metab. Brain Dis..

[71] Zhang J., Li M., Zhao T., Cao J., Liu Y., Wang Y., Wang Y., Cheng G. (2022). E se tea alleviates acetaminophen-induced liver injury by activating the nrf2 signaling pathway. Food Funct..

[72] Lee J., Jung E., Kim Y.S., Park D., Toyama K., Date A., Lee J. (2013). Phloridzin isolated from acanthopanax senticosus promotes proliferation of alpha6 integrin (cd 49f) and beta1 integrin (cd29) enriched for a primary keratinocyte population through the erk-mediated mtor pathway. Arch. Dermatol. Res..

[73] Antika L.D., Lee E., Kim Y., Kang M., Park S., Kim D.Y., Oh H., Choi Y., Kang Y. (2017). Dietary phlorizin enhances osteoblastogenic bone formation through enhancing -catenin activity via gsk-3 inhibition in a model of senile osteoporosis. J. Nutr. Biochem..

[74] Yamazaki Y., Harada S., Wada T., Yoshida S., Tokuyama S. (2016). Sodium transport through the cerebral sodium-glucose transporter exacerbates neuron damage during cerebral ischaemia. J. Pharm. Pharmacol..

[75] Harada S., Yamazaki Y., Nishioka H., Tokuyama S. (2013). Neuroprotective effect through the cerebral sodium-glucose transporter on the development of ischemic damage in global ischemia. Brain Res..

[76] Kashiwagi Y., Nagoshi T., Yoshino T., Tanaka T.D., Ito K., Harada T., Takahashi H., Ikegami M., Anzawa R., Yoshimura M. (2015). Expression of sglt1 in human hearts and impairment of cardiac glucose uptake by phlorizin during ischemia-reperfusion injury in mice. PLoS ONE.

[77] Hirose M., Shibazaki T., Nakada T., Kashihara T., Yano S., Okamoto Y., Isaji M., Matsushita N., Taira E., Yamada M. (2014). Phlorizin prevents electrically-induced ventricular tachyarrhythmia during ischemia in langendorff-perfused guinea-pig hearts. Biol. Pharm. Bull..

[78] Abouelenein D., Caprioli G., Mustafa A.M. (2023). Phloridzin: Advances on resources, biosynthetic pathway, bioavailability, bioactivity, and pharmacology. Handbook of Dietary Flavonoids.

[79] Ehrenkranz J.R., Lewis N.G., Ronald Kahn C., Roth J. (2005). Phlorizin: A review. Diabetes/Metab. Res. Rev..

[80] Fernando W., Clark R.F., Rupasinghe H., Hoskin D.W., Coombs M. (2023). Phloridzin docosahexaenoate inhibits spheroid formation by breast cancer stem cells and exhibits cytotoxic effects against paclitaxel-resistant triple negative breast cancer cells. Int. J. Mol. Sci..

[81] Fernando W., Coyle K., Marcato P., Vasantha R.H., Hoskin D.W. (2019). Phloridzin docosahexaenoate, a novel fatty acid ester of a plant polyphenol, inhibits mammary carcinoma cell metastasis. Cancer Lett..

[82] Fernando W., Coombs M., Hoskin D.W., Rupasinghe H. (2016). Docosahexaenoic acid-acylated phloridzin, a novel polyphenol fatty acid ester derivative, is cytotoxic to breast cancer cells. Carcinogenesis.

